# Effects of immersive virtual reality on anxiety, depression, cancer-related fatigue, and quality of life in cancer patients undergoing chemotherapy: a systematic review and meta-analysis

**DOI:** 10.3389/fonc.2026.1796508

**Published:** 2026-04-13

**Authors:** Yuan Gao, Xinyue Liu, Xi Liao, Jie Yang

**Affiliations:** 1 Colorectal Cancer Center, West China Hospital, Sichuan University, Chengdu, Sichuan, China; 2 Department of General Surgery, West China Hospital, Sichuan University, Chengdu, Sichuan, China

**Keywords:** cancer, chemotherapy, immersive virtual reality, meta-analysis, non-pharmacological intervention

## Abstract

**Background:**

Cancer patients undergoing chemotherapy usually experience a variety of adverse symptoms, such as anxiety, depression, and cancer-related fatigue, which negatively impact their quality of life. Immersive Virtual Reality (IVR) is considered a promising non-pharmacological intervention for cancer patients undergoing chemotherapy. However, the effects of IVR on anxiety, depression, cancer-related fatigue, and quality of life in adult cancer patients undergoing chemotherapy remain unclear.

**Methods:**

A comprehensive search was conducted across eleven electronic databases, including PubMed, Web of Science, Scopus, Embase, Cochrane Library, CINAHL, PsycINFO, CNKI, WanFang, VIP, and CBM, from their inception to January 2026. We included randomized controlled trials examining the impact of IVR on anxiety, depression, cancer-related fatigue, and quality of life among adult cancer patients undergoing chemotherapy. The methodological quality of the included studies was assessed using the Cochrane Risk of Bias tool 2.0 (ROB 2.0). The certainty of the evidence was graded using the Grading of Recommendations, Assessment, Development and Evaluation (GRADE) framework. For quantitative synthesis, a random-effects model was applied to all meta-analyses.

**Results:**

A total of 11 randomized controlled trials involving 1160 cancer patients undergoing chemotherapy were included. The meta-analysis results demonstrated that IVR significantly reduced anxiety (SMD = -1.02, 95% CI: -1.70 to -0.33, low certainty evidence), depression (SMD = -1.59, 95% CI: -2.71 to -0.46, low certainty evidence), and cancer-related fatigue (SMD = -1.17, 95% CI: -1.96 to -0.37, low certainty evidence), as well as improved quality of life (SMD = 0.76, 95% CI: 0.32 to 1.21, low certainty evidence) compared to usual care.

**Conclusions:**

Compared with usual care, IVR appears to be more effective in reducing anxiety, depression, and cancer-related fatigue in cancer patients undergoing chemotherapy, while also improving quality of life. However, these findings should be validated by more high-quality randomized controlled trials.

## 1 Introduction

Chemotherapy remains one of the most well-established systemic treatments for a wide range of cancers (1). It is estimated that the global annual incidence of cancer patients receiving chemotherapy is projected to increase by 53% between 2018 and 2040, from 9.8 million to 15.0 million (2). Despite its efficacy in suppressing tumors, chemotherapy usually triggers considerable adverse physiological (cancer-related fatigue, nausea, vomiting, gastrointestinal disturbances, and neuropathy) and psychological symptoms (anxiety and depression), thereby impairing the quality of life for cancer patients (3–6). A high prevalence of anxiety (42.6%) and depression (40.9%) has been reported among patients during chemotherapy (4). Furthermore, a meta-analysis revealed that 52.5% of cancer patients during chemotherapy experienced moderate to severe fatigue (5). It is noteworthy that the quality of life of cancer patients has been observed to progressively decline during chemotherapy (6). Therefore, implementing alternative or complementary therapies to reduce adverse psychological and physiological experiences in cancer patients undergoing chemotherapy and achieve desired health outcomes has become a priority in oncology care.

Immersive Virtual Reality (IVR) is a three-dimensional virtual environment created through smart devices (computers, virtual reality headsets, smartphones), delivering an immersive experience to users via sensory feedback channels such as sight and sound (7). IVR is considered a promising alternative or complementary therapy in the healthcare field, particularly for cancer patients undergoing chemotherapy (8). Owing to its immediacy, convenience, and entertainment value, IVR holds particular potential in chemotherapy settings (9). Specifically, IVR enables bedside interventions during infusion or chemotherapy breaks. By redirecting attention away from infusion-related prompts and internal discomfort, and replacing the clinical environment with an engaging, controllable virtual setting, it breaks the inherent negative sensory and cognitive feedback loop associated with chemotherapy (10). However, several studies on the efficacy of IVR for cancer patients undergoing chemotherapy have drawn sharply contrasting conclusions. A previous study conducted in Italy demonstrated that IVR alleviated anxiety, depression, and cancer-related fatigue in patients undergoing chemotherapy (11). Yet these findings have not been supported by findings from other similar studies (12, 13). Therefore, the findings highlight the necessity of rigorous data synthesis to draw reliable conclusions that could guide clinical practice.

To date, only two meta-analyses have quantitatively synthesized the evidence on the effects of IVR on cancer patients undergoing chemotherapy (14, 15). However, both studies have significant limitations. Firstly, their outcome measures focused primarily on anxiety, depression, and cancer-related fatigue, while failing to critically evaluate quality of life. Secondly, the number of studies included was very limited, and the pooled sample sizes for individual outcomes were small. This may have compromised the reliability of their conclusions due to limited statistical power. Finally, no sensitivity analysis was conducted on the pooled results, undermining the credibility of their conclusions. Therefore, this systematic review and meta-analysis aimed to determine the effects of IVR on anxiety, depression, cancer-related fatigue, and quality of life specifically in cancer patients undergoing chemotherapy.

## 2 Materials and methods

This study was conducted following the Preferred Reporting Items for Systematic Reviews and Meta-Analyses (PRISMA) guidelines (16) and registered with PROSPERO (CRD420261290635).

### 2.1 Search strategy

A comprehensive search of eleven electronic databases, including PubMed, Web of Science, Scopus, Embase, Cochrane Library, CINAHL, PsycINFO, China National Knowledge Infrastructure (CNKI), WanFang, VIP, and SinoMed (CBM), was conducted. The search spanned records from inception to January 2026, integrating relevant MeSH terms and synonyms with Boolean operators. Search terms comprised “neoplasms,” “cancer,” “chemotherapy,” “immersive virtual reality,” and “randomized clinical trial.” To identify further potentially eligible studies, the reference lists of all included publications were screened. The detailed search strategies are provided in **Supplementary Table S1**.

### 2.2 Eligibility criteria

Articles were selected using the PICOS criteria: (1) Population: adult cancer patients undergoing chemotherapy; (2) Intervention: using IVR during chemotherapy; (3) Comparison: usual care; (4) Outcomes: anxiety, depression, cancer-related fatigue, and quality of life, with at least one of these outcomes being assessed and reported; (5) Study design: randomized controlled trials. Studies were excluded if they were duplicate publications, non-empirical studies (conference abstracts, case reports, protocols, reviews), contained insufficient data for meta-analysis, or were published in languages other than English or Chinese.

### 2.3 Data extraction

Data extraction was performed independently by two reviewers using a standardized form. Any discrepancies between reviewers were resolved through consultation with a third reviewer to reach a consensus. The extracted data included: first author, publication year, country, participants’ characteristics (sample size, mean age, and cancer type), experimental group characteristics (main content, length, and timing), control group, and outcome measures.

### 2.4 Quality assessment and certainty of evidence

Two reviewers independently assessed both the methodological quality of included studies and the certainty of evidence. Disagreements were resolved by consulting a third reviewer. Methodological quality was appraised using the Cochrane Risk of Bias Tool 2.0 (ROB 2.0) (17), which evaluates five bias categories: randomization process, deviations from intended interventions, missing outcome data, outcome measurement, and selection of the reported result. Each study was determined to be at “low,” “some concerns,” or “high” risk of bias.

The certainty of evidence for all outcomes (anxiety, depression, cancer-related fatigue, and quality of life) was evaluated according to the Grading of Recommendations, Assessment, Development, and Evaluations (GRADE) framework (18). Following GRADE guidelines, the evidence for each outcome was rated as “high,” “moderate,” “low,” or “very low” based on the following domains: risk of bias, inconsistency, indirectness, imprecision, and publication bias.

### 2.5 Statistical analysis

Data analysis was conducted using Stata 17.0 software. As all outcome measures were continuous variables assessed using different instruments, the standardized mean difference (SMD) with 95% confidence intervals (CI) was considered the effect size for the meta-analysis. Heterogeneity was evaluated using the I² statistic (<25%, low; 25-50%, moderate; >50%, high). A fixed-effects model was applied to calculate the pooled results when the observed heterogeneity was low. Otherwise, a random-effects model was used. The robustness of pooled results was evaluated using a leave-one-out sensitivity analysis. Potential sources of heterogeneity were examined through subgroup analyses stratified by intervention content and length. All tests were two-sided, and P<0.05 was considered statistically significant.

## 3 Results

### 3.1 Literature selection

**Figure 1:**
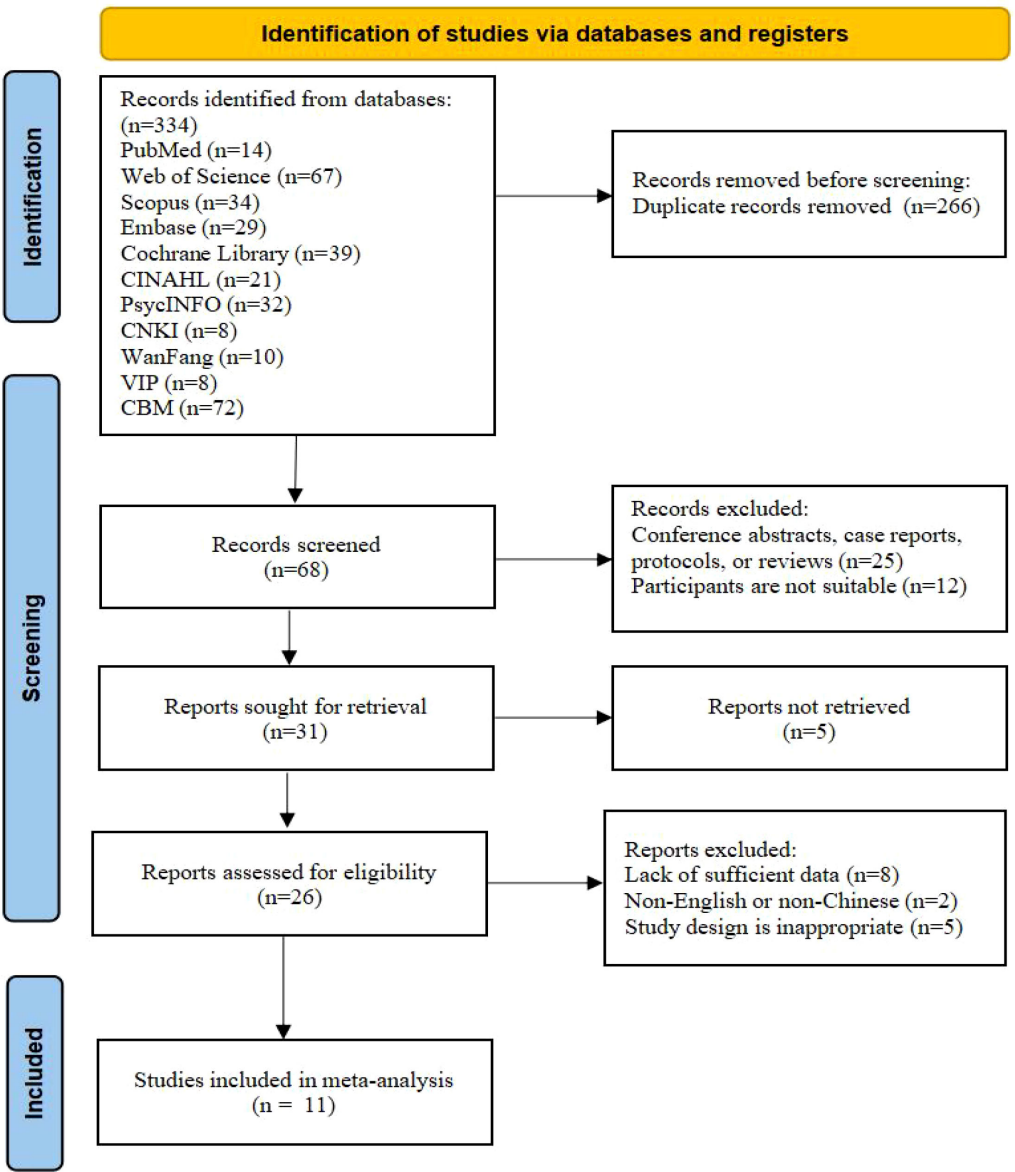
Flow diagram of study selection.

A total of 334 records were initially identified. Following the removal of duplicates, 68 articles were screened by title and abstract, of which 31 proceeded to full-text review for eligibility. After a detailed assessment, 11 studies were ultimately included in the meta-analysis, as illustrated in **Figure 1**.

### 3.2 Study characteristics

The characteristics of the included studies are detailed in **Table 1**. The 11 included studies, published between 2007 and 2025, comprised a total of 1160 cancer patients undergoing chemotherapy. The mean age of participants across the included studies ranged from 33.5 to 72.9 years. Most of the participants suffered from breast cancer, while others had ovarian cancer, lung cancer, leukemia, and other solid cancers. Sample sizes across the studies ranged from 44 to 327 participants. Moreover, 7 trials were conducted in China, 2 in Italy, 1 in Turkey, and 1 in the USA.

**Table 1:** Characteristics of included studies.

Study	Country	Participants				Experimental group (VR)		Outcomes (instrument)
		Sample size	Mean age	Cancer type	Main content	Length	Timing	
Chirico et al., 2020 (11)	Italy	EG: 30 CG: 34	EG: 55.18 ± 5.7 CG: 56.20 ± 6.79	Breast cancer	The intervention utilized head-mounted device to deliver immersive, relaxing natural scenarios. These included exploring an island, walking through a forest, observing wildlife, climbing a mountain, and swimming in the sea.	20 minutes	During chemotherapy	Anxiety (SAI) Depression (POMS) Fatigue (POMS)
Fabi et al., 2022 (12)	Italy	EG: 22 CG: 22	NA	Breast and ovarian cancer	Using the standalone headset, patients under operator supervision autonomously selected and viewed relaxing 360° videos, with content curated for comfort and immersion.	60–90 minutes	During chemotherapy	Anxiety (STAI) Quality of life (EORTC QLQ-C30)
Fu and Qu. 2025 (19)	China	EG: 61 CG: 61	EG: 72.5 ± 6.2 CG: 72.9 ± 6.1	Breast cancer	An 8-week mindfulness intervention using a virtual reality system was conducted, which included mindfulness education and a progressive training program consisting of six modules such as seated meditation, body scanning, and mindfulness yoga.	60 minutes	During chemotherapy	Fatigue (CRF) Quality of life (FACT-B)
Guo et al., 2025 (20)	China	EG: 30 CG: 30	EG: 62.53 ± 6.50 CG: 62.80 ± 7.29	Lung cancer	Carrying out psychosomatic interaction training in a virtual environment constructed with photos of natural scenes such as oceans and forests.	10 minutes	During chemotherapy	Anxiety (SAI) Depression (BDI)
Lei et al., 2021 (21)	China	EG: 30 CG: 30	NA	Mixed cancer	Participants wore head-mounted devices to experience a relaxing virtual environment.	30 minutes	During chemotherapy	Fatigue (PFS)
Li et al., 2024 (22)	China	EG: 163 CG: 164	EG: 53.60 ± 9.40 CG: 55.20 ± 9.70	Breast cancer	Participants experience traversing forests, walking along beaches, sightseeing at sea, or engaging in relaxation exercises guided by voice instructions.	15–20 minutes	During chemotherapy	Quality of life (EORTC QLQ-C30)
Schneider and Hood. 2007 (13)	USA	EG: 106 CG: 104	53.97 ± 10.89	Mixed cancer	Using the head-mounted display, participants experienced scenarios including deep sea diving, walking through an art museum, exploring ancient worlds, and solving a mystery.	45–90 minutes	During chemotherapy	Anxiety (SAI) Fatigue (PFS)
Uslu et al., 2023 (23)	Turkey	EG: 33 CG: 33	EG: 53.42 ± 9.25 CG: 51.42 ± 8.53	Breast cancer	Participants watch their favorite 360-degree nature videos through virtual reality glasses.	30 minutes	During chemotherapy	Anxiety (STAI) Fatigue (CFS)
Zhang et al., 2022 (24)	China	EG: 38 CG: 39	EG: 52.29 ± 57.69 CG: 51.03 ± 7.98	Breast cancer	Using head-mounted VR devices, patients are immersed in natural scenes to achieve mind-body regulation through audiovisual interaction.	30 minutes	During chemotherapy	Anxiety (SAS) Depression (SDS) Fatigue (PFS) Quality of life (FACT-B)
Zhang et al., 2024 (25)	China	EG: 30 CG: 30	EG: 33.50 ± 11.06 CG: 35.27 ± 10.55	Leukemia	Participants immerse themselves in environments such as forests or beaches through VR devices for meditation.	20 minutes	During chemotherapy	Anxiety (SAI) Depression (CES-D) Quality of life (FACT-Leu)
Zhao and Xu. 2021 (26)	China	EG: 35 CG: 35	EG: 51.45 ± 8.46 CG: 51.37 ± 8.52	Lung cancer	Carrying out cognitive training through the imitation of the movements of virtual characters (such as Tai Chi, rowing)	60 minutes	During chemotherapy	Anxiety (HAMA) Depression (HAMD) Quality of life (EORTC QLQ-C30)

### 3.3 Risk of bias assessment results

**Figure 2:**
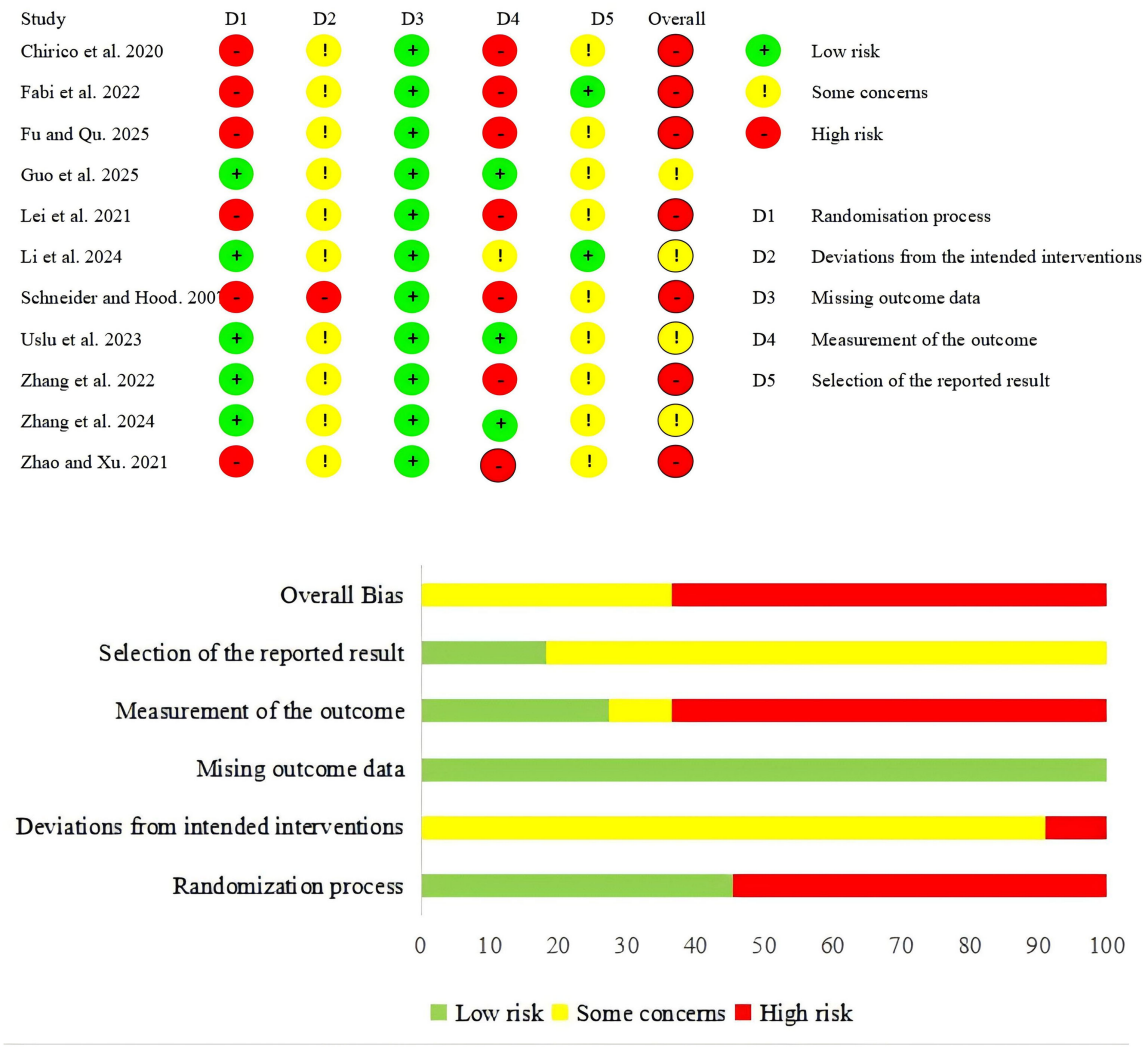
Summary of risk of bias.

The methodological quality of the included studies is presented in **Figure 2**. Overall, no studies were deemed to be low risk for bias, four were rated as having some concerns, and 7 were at high risk of bias. Domain-specific assessments revealed that for the randomization process, 5 studies were at low risk and 6 at high risk. Regarding deviations from intended interventions, 10 studies raised some concerns, and 1 was at high risk. All studies were judged as low risk for missing outcome data. In the measurement of the outcome, 3 studies were at low risk, 1 raised some concerns, and 7 were at high risk. For the selection of the reported result, 2 studies were at low risk, and 9 raised some concerns.

### 3.4 Main outcomes

#### 3.4.1 Anxiety

**Figure 3:**
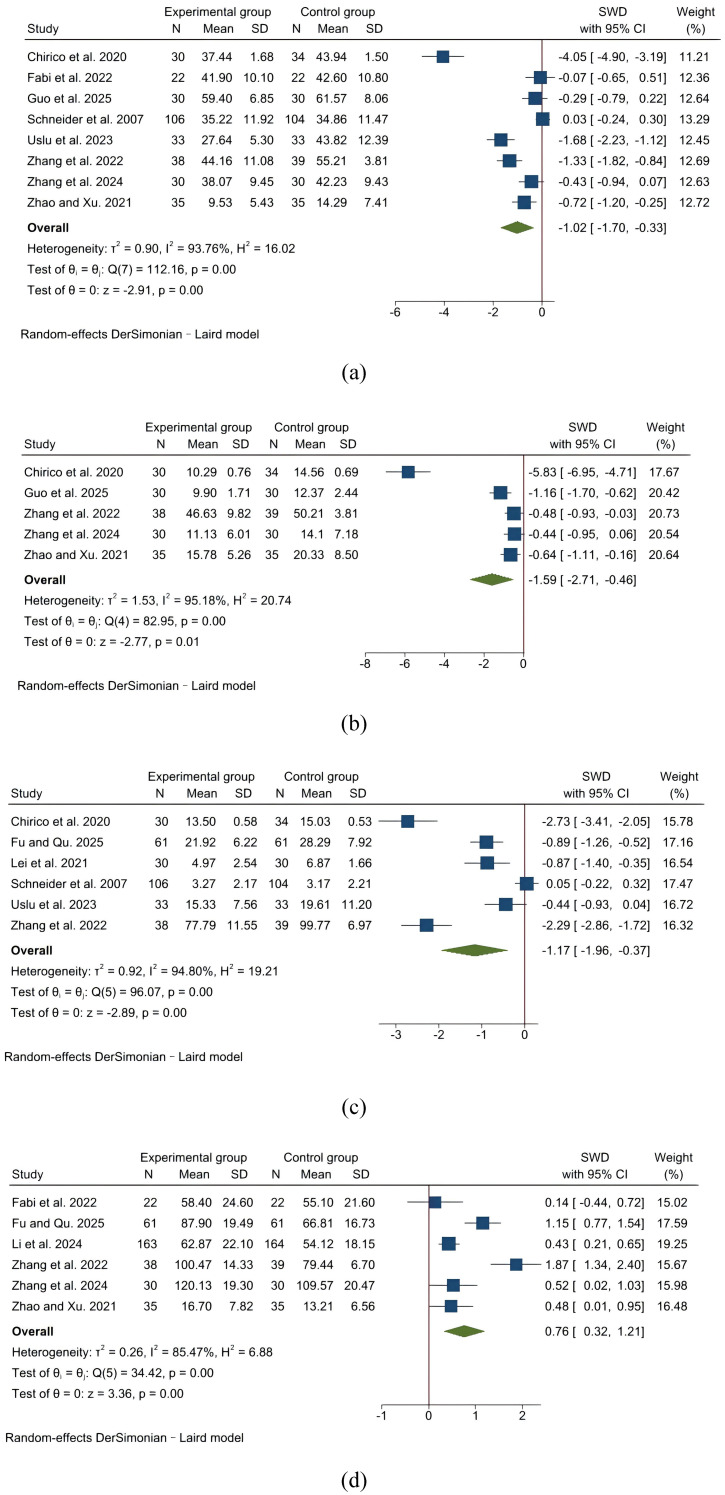
The effects of IVR on anxiety, depression, cancer-related fatigue, and quality of life in cancer patients undergoing chemotherapy. **(a)** anxiety; **(b)** depression; **(c)** cancer-related fatigue; **(d)** quality of life.

8 studies provided sufficient data on the effect of IVR on anxiety in cancer patients undergoing chemotherapy. As shown in **Figure 3a**, the pooled data demonstrated a statistically significant reduction in anxiety among adult cancer patients undergoing chemotherapy receiving IVR compared to usual care (SMD = -1.02, 95% CI: -1.70 to -0.33; I² = 94%), indicating a substantial effect size. No significant difference was observed between psychology-integrated IVR and distraction-only IVR in their effects on anxiety levels among cancer patients receiving chemotherapy (**Supplementary Figure S1a**).

In addition, subgroup analysis showed that a single IVR session lasting ≤30 minutes significantly reduced anxiety among cancer patients undergoing chemotherapy more effectively than sessions lasting >30 minutes (**Supplementary Figure S1b**).

#### 3.4.2 Depression

A total of 5 trails reported the data on the effectiveness of IVR on depression among cancer patients undergoing chemotherapy. The pooled analysis showed that IVR was associated with a statistically significant reduction in depression level compared to usual care among adult cancer patients undergoing chemotherapy (SMD = -1.59, 95% CI: -2.71 to -0.46), despite substantial heterogeneity (I² = 95%; **Figure 3b**). Subgroup analyses revealed no significant differences in the alleviation of depression in cancer patients undergoing chemotherapy based on variations in IVR content or single-session length (**Supplementary Figure S2**).

#### 3.4.3 Cancer-related fatigue

In the included studies, 6 trials evaluated the impact of IVR on cancer-related fatigue in patients undergoing cancer chemotherapy. Meta-analysis results indicated that cancer patients undergoing chemotherapy who received IVR reported and experienced lower levels of cancer-related fatigue compared to those receiving usual care (SMD = -1.17, 95% CI: -1.96 to -0.37; I² = 95%; **Figure 3c**). Subgroup analyses failed to find a statistically significant difference in the effect of IVR content or single-session length on alleviating cancer-related fatigue among cancer patients undergoing chemotherapy (**Supplementary Figure S3**).

#### 3.4.4 Quality of life

In the included studies, 6 trials assessed the impact of IVR on quality of life among patients undergoing cancer chemotherapy. A meta-analysis revealed that IVR was related to a significant improvement in quality of life compared to usual care in patients undergoing chemotherapy (SMD = 0.76, 95% CI: 0.32 to 1.21; I² = 85%; **Figure 3d**). Subgroup analyses demonstrated that the efficacy of IVR in improving quality of life in cancer patients undergoing chemotherapy did not differ significantly based on IVR content or single-session length (**Supplementary Figure S4**).

#### 3.5 Sensitivity analyses

Sensitivity analyses for anxiety, depression, cancer-related fatigue, and quality of life demonstrated the robustness of the pooled results (**Supplementary Figure S5**). Specifically, after excluding each study individually, the pooled results for each outcome showed no significant changes.

#### 3.6 The certainty of evidence

According to the GRADE framework, the certainty of evidence for the effectiveness of IVR in reducing anxiety, depression, and fatigue, as well as improving quality of life in cancer patients undergoing chemotherapy, was rated as low. Importantly, none of the analyzed studies were graded as overall having a low risk of bias. This rating, along with the supporting rationale, is detailed in **Supplementary Table S2**.

## 4 Discussion

In this meta-analysis encompassing 11 randomized controlled trials involving 1160 cancer patients undergoing chemotherapy, the effects of IVR on anxiety, depression, cancer-related fatigue, and quality of life were synthesized. Specifically, compared to usual care, IVR was effective in reducing anxiety, depression, and cancer-related fatigue, while improving quality of life in cancer patients undergoing chemotherapy. However, given the low certainty of evidence for the primary outcomes, more high-quality randomized controlled trials remain necessary to draw more reliable conclusions.

Anxiety and depression are prevalent unpleasant emotional states experienced by cancer patients undergoing chemotherapy (4). Our findings demonstrated that IVR may significantly reduce anxiety and depression levels in cancer patients undergoing chemotherapy, consistent with the results of previous meta-analyses. The Conservation of Resources Theory posits that the perception of threatened or depleted psychological resources directly leads to negative emotional states (27). During chemotherapy, the pain, nausea, and vomiting caused by treatment exhaust patients’ mental resources, leading them to remain fixated on their suffering and fears, which in turn triggers anxiety and depression (28). By immersing participants in environments such as beaches or oceans, IVR acts as an effective distraction. This provides temporary relief from real-world stressors and promotes psychosomatic relaxation, generating positive psychological responses that alleviate symptoms of anxiety and depression (11). Moreover, when attention is directed toward a tranquil beach, an enchanted forest, or engaging interactive games, the cognitive resources available for managing anxiety and depression are significantly reduced (19). It is worth noting that several IVR platforms also incorporate psychological intervention techniques such as mindfulness meditation and cognitive behavioral therapy (19, 20). These approaches directly induce relaxation and positive feelings in patients, effectively replenishing depleted psychological resources and reducing negative emotions. Notably, our subgroup analysis revealed that single IVR sessions lasting ≤30 minutes were more effective in reducing anxiety than sessions exceeding 30 minutes. Although this finding is somewhat counterintuitive, it may be explained by the cognitive demands of the chemotherapy context. Patients undergoing chemotherapy often experience significant fatigue and reduced attention spans (5). Extended IVR sessions, despite their immersive quality, might inadvertently lead to mental fatigue or information overload, diminishing their anxiolytic benefits. In contrast, shorter, focused sessions may provide an optimal dose of distraction without depleting the patient’s limited attentional capacity. This suggests that beyond a certain threshold, the therapeutic benefit of a single VR session may plateau or even decline. Moreover, this difference may also be driven by inherent bias and lack of power in the analyzed trials, as all trials with interventions of less than or equal to 30 minutes had sample sizes of fewer than 40 participants, and half were graded as having a high risk of bias. However, given the exploratory nature of this subgroup analysis and the variability in intervention protocols across studies, this finding warrants cautious interpretation and further investigation.

Cancer-related fatigue is one of the most distressing symptoms for cancer patients undergoing chemotherapy (5). This meta-analysis demonstrated that IVR was associated with a significant reduction in cancer-related fatigue among cancer patients undergoing chemotherapy. Cancer-related fatigue generally presents as a persistent, subjective sense of exhaustion or a decline in physical functioning, affecting physical, emotional, sensory, and cognitive capacities (29). In an IVR environment, the patient’s parasympathetic nervous response is activated, inducing a relaxation reaction manifested by decreased heart rate, lowered blood pressure, and reduced levels of stress hormones (26). The physiological relaxation directly counteracts the physical and mental exhaustion that accompanies chronic stress. Positive IVR experiences stimulate the brain to release endorphins and dopamine. This not only improves mood but may also directly boost energy levels and motivation, alleviating fatigue (30). Additionally, IVR provides structured, engaging cognitive training through tasks such as exploration and puzzle-solving. This simple cognitive exercise in low-stress conditions helps alleviate symptoms of cognitive fatigue, including mental fog and difficulty concentrating (21).

Improving the quality of life for chemotherapy patients remains one of the primary goals in cancer care. Our study revealed that IVR significantly improved the quality of life for cancer patients undergoing chemotherapy. Quality of life of cancer patients is a multidimensional construct encompassing several core domains, including physical, role, social, emotional, and cognitive functioning (31). First, nature and landscape-based IVR enables patients to engage in immersive experiences of biological environments, promoting emotional well-being and reducing fatigue (24). Furthermore, the unique IVR experience itself serves as a conversation topic for patients to share with family and friends, thereby fostering social connections (32). Finally, game-based or exploration-based IVR requires a moderate level of navigation, decision-making, or problem-solving skills, providing low-risk cognitive exercise for patients experiencing cognitive decline (21). Therefore, IVR promotes an improvement in the quality of life for cancer patients undergoing chemotherapy by directly enhancing multiple core domains that constitute quality of life.

Several limitations should be considered. First, a potential limitation is the inclusion of both English and Chinese studies, with a significant proportion (7 of 11 RCTs) originating from China. This geographic concentration may introduce publication and language bias, limiting the generalizability of the findings to other populations with different backgrounds and healthcare systems. Second, only 11 studies with limited sample sizes were ultimately included, which may limit the statistical power and generalizability of our findings. Third, substantial heterogeneity was observed across the pooled outcomes, attributable to variations in participant characteristics (cancer type, disease stage, treatment regimen), intervention protocols (content, duration, frequency), and outcome assessment methods (diverse instruments and recall periods). Furthermore, differences in methodological quality and risk of bias across the included studies may also have contributed to the observed heterogeneity, as studies with inadequate randomization concealment or lack of blinding might systematically overestimate treatment effects. However, subgroup analyses did not adequately explain the sources of heterogeneity, likely due to the limited number of studies within each subgroup and the inconsistent reporting of key moderators across studies. This unexplained heterogeneity represents a significant limitation of the current evidence base and highlights the need for future research to adopt standardized outcome measures and rigorous study designs. Fourth, critical clinical characteristics such as gender, disease stage, and line of chemotherapy were inconsistently reported across the included studies and could not be incorporated into our analyses. These variables may moderate the effectiveness of IVR, and their absence limits our ability to identify specific patient subgroups that might derive greater benefit. Finally, our findings should be interpreted as suggestive rather than definitive in light of the low certainty of evidence as assessed by the GRADE framework. The low certainty is further compounded by the lack of long-term follow-up data, which prevents assessment of the sustained clinical impact of IVR.

## 5 Conclusions

In conclusion, this meta-analysis demonstrated that IVR had the potential to alleviate anxiety, depression, and cancer-related fatigue, and improve quality of life in cancer patients undergoing chemotherapy. However, the observed heterogeneity and the low certainty of the available evidence collectively limit the strength of conclusions and necessitate further well-designed randomized controlled trials to establish more robust evidence. As such, data in support of the utility of IVR in this space should only be considered hypothesis-generating, and will remain so until the quality of investigative trials improves.
