# Supplementary material for: Effects of immersive virtual reality on anxiety, depression, cancer-related fatigue, and quality of life in cancer patients undergoing chemotherapy: a systematic review and meta-analysis
Source: Front Oncol. 2026 Apr 13;16:1796508. doi: 10.3389/fonc.2026.1796508 (PMC13111075; doi:10.3389/fonc.2026.1796508)
Supplement: Supplementary file 1 [file Supplementaryfile1.docx]

**Supplemental Materials**

**Table S1.** The search strategies

| **Databases** | **Step** | **Search Strategies** |
| --- | --- | --- |
| PubMed | #1 | "[neoplasms](https://www.ncbi.nlm.nih.gov/mesh/68009369)"[Mesh Terms] Sort by: Best match |
|  | #2 | "cancer"[Title/Abstract] OR "tumor"[Title/Abstract] OR "tumour"[Title/Abstract] OR "carcinoma"[Title/Abstract] OR "malignancy"[Title/Abstract] Sort by: Best match |
|  | #3 | "chemotherapy"[Title/Abstract] OR "[drug therapy](https://www.ncbi.nlm.nih.gov/mesh/81000188)"[Mesh Terms] OR "[antineoplastic agents](https://www.ncbi.nlm.nih.gov/mesh/68000970)"[Mesh Terms] Sort by: Best match |
|  | #4 | "immersive virtual reality"[Title/Abstract] OR "virtual reality"[Title/Abstract] OR "head-mounted display"[Title/Abstract] Sort by: Best match |
|  | #5 | "RCT"[Title/Abstract] OR "randomized clinical trial"[Title/Abstract] OR "randomized controlled trial"[Title/Abstract] OR "randomized trial"[Title/Abstract] OR "randomised controlled trial"[Title/Abstract] OR "randomised trial"[Title/Abstract] Sort by: Most Recent |
|  | #6 | #1 OR #2 Sort by: Best match |
|  | #7 | #3 AND #4 AND #5 AND #6 Sort by: Best match |
| Web of Science | #1 | **TS=(**[neoplasms](https://www.ncbi.nlm.nih.gov/mesh/68009369)**) OR TS=(cancer**) OR TS=(tumor) OR TS=(tumour) OR TS=(carcinoma) OR TS=(malignancy) |
|  | #2 | **Ts=(**chemotherapy**) OR Ts=(**[drug therapy](https://www.ncbi.nlm.nih.gov/mesh/81000188)**) OR** TS=([antineoplastic agents](https://www.ncbi.nlm.nih.gov/mesh/68000970)) |
|  | #3 | TS=(immersive virtual reality) OR **TS=(**virtual reality**) OR TS=(**head-mounted display) |
|  | #4 | TS=(RCT) OR **TS=(**randomized clinical trial**) OR TS=(**randomized controlled trial) OR TS=(randomized trial) OR TS=(randomised controlled trial) OR TS=(randomised trial) |
|  | #5 | #1 AND #2 AND #3 AND #4 |
| Scopus | #1 | ABS("[neoplasms](https://www.ncbi.nlm.nih.gov/mesh/68009369)" OR "cancer" OR "tumor" OR "tumour" OR "carcinoma" OR "malignancy") |
|  | #2 | ABS("chemotherapy" OR "[drug therapy](https://www.ncbi.nlm.nih.gov/mesh/81000188)" OR "[antineoplastic agents](https://www.ncbi.nlm.nih.gov/mesh/68000970)") |
|  | #3 | ABS ("immersive virtual reality" OR "virtual reality" OR "head-mounted display") |
|  | #4 | ABS ("RCT" OR "randomized clinical trial" OR "randomized controlled trial" OR "randomized trial" OR "randomised controlled trial OR "randomised trial) |
|  | #5 | #1 AND #2 AND #3 AND #4 |
| Embase | **#1** | **'**[neoplasms](https://www.ncbi.nlm.nih.gov/mesh/68009369)**'/exp** |
|  | **#2** | 'cancer':ti,ab,kw OR 'tumor':ti,ab,kw OR 'tumour':ti,ab,kw OR 'carcinoma':ti,ab,kw OR 'malignancy':ti,ab,kw |
|  | **#3** | **'**chemotherapy**'/exp** |
|  | **#4** | '[drug therapy](https://www.ncbi.nlm.nih.gov/mesh/81000188)**':ti,ab,kw OR '**[antineoplastic agents](https://www.ncbi.nlm.nih.gov/mesh/68000970)**':ti,ab,kw** |
|  | **#5** | 'immersive virtual reality**':ti,ab,kw OR '**virtual reality**':ti,ab,kw OR '**head-mounted display**':ti,ab,kw** |
|  | #6 | 'RCT**':ti,ab,kw OR '**randomized clinical trial**':ti,ab,kw OR '**randomized controlled trial**':ti,ab,kw OR '**randomized trial**':ti,ab,kw OR '**randomised controlled trial**':ti,ab,kw OR '**randomised trial**':ti,ab,kw** |
|  | #7 | #1 OR #2 |
|  | #8 | #3 OR #4 |
|  | #9 | #5 AND #6 AND #7 AND #8 |
| Cochrane Library | #1 | MeSH descriptor: [Neoplasms] explode all trees |
|  | #2 | (cancer):ti,ab,kw OR (tumor):ti,ab,kw OR (tumour):ti,ab,kw OR (carcinoma):ti,ab,kw OR (malignancy):ti,ab,kw |
|  | #3 | MeSH descriptor: [[Drug Therapy](https://www.ncbi.nlm.nih.gov/mesh/81000188)] explode all trees |
|  | #4 | (immersive virtual reality)**:ti,ab,kw OR (**virtual reality)**:ti,ab,kw OR (**head-mounted display)**:ti,ab,kw** |
|  | #5 | (RCT)**:ti,ab,kw OR (**randomized clinical trial)**:ti,ab,kw OR (**randomized controlled trial)**:ti,ab,kw OR (**randomized trial)**:ti,ab,kw OR (**randomised controlled trial)**:ti,ab,kw OR (**randomised trial)**:ti,ab,kw** |
|  | #6 | #1 OR #2 |
|  | #8 | #3 AND #4 AND #5 AND #6 |
| CINAHL | S1 | **TI** [neoplasms](https://www.ncbi.nlm.nih.gov/mesh/68009369) **OR TI cancer** OR TI tumor OR TI tumour OR TI carcinoma OR TI malignancy |
|  | S2 | **TI** chemotherapy **OR TI** [drug therapy](https://www.ncbi.nlm.nih.gov/mesh/81000188) **OR** TI [antineoplastic agents](https://www.ncbi.nlm.nih.gov/mesh/68000970) |
|  | S3 | TI immersive virtual reality **OR TI** virtual reality **OR TI** head-mounted display |
|  | S4 | TI RCT **OR TI** randomized clinical trial **OR TI** randomized controlled trial **OR TI** randomized trial **OR TI** randomised controlled trial **OR TI** randomised trial |
|  | S5 | S1 AND S2 AND S3 AND S4 |
| PsycINFO | S1 | **TI** [neoplasms](https://www.ncbi.nlm.nih.gov/mesh/68009369) **OR TI cancer** OR TI tumor OR TI tumour OR TI carcinoma OR TI malignancy |
|  | S2 | **TI** chemotherapy **OR TI** [drug therapy](https://www.ncbi.nlm.nih.gov/mesh/81000188) **OR** TI [antineoplastic agents](https://www.ncbi.nlm.nih.gov/mesh/68000970) |
|  | S3 | TI immersive virtual reality **OR TI** virtual reality **OR TI** head-mounted display |
|  | S4 | TI RCT **OR TI** randomized clinical trial **OR TI** randomized controlled trial **OR TI** randomized trial **OR TI** randomised controlled trial **OR TI** randomised trial |
|  | S5 | S1 AND S2 AND S3 AND S4 |
| CNKI |  | (SU="肿瘤"+ "恶性肿瘤" + "癌症") AND (SU="化疗" + "化学治疗"+ "辅助化疗") AND (SU="沉浸式虚拟现实" + "虚拟现实") |
| WanFang |  | 题名或关键词:(肿瘤 OR 恶性肿瘤 OR 癌症) and 题名或关键词:(化疗 OR 化学治疗 OR 辅助化疗) and 题名或关键词:(沉浸式虚拟现实 OR 虚拟现实) |
| VIP |  | (题名或关键词=肿瘤 OR 恶性肿瘤OR 癌症) and (题名或关键词=化疗 OR 化学治疗 OR 辅助化疗) and (题名或关键词=沉浸式虚拟现实 OR 虚拟现实) |
| CBM |  | ("肿瘤"[标题] OR "恶性肿瘤"[标题] OR "癌症"[标题] ) AND ( "化疗"[标题] OR "化学治疗"[标题] OR "辅助化疗"[标题]) AND ( "沉浸式虚拟现实"[标题] OR "虚拟现实"[标题]) |


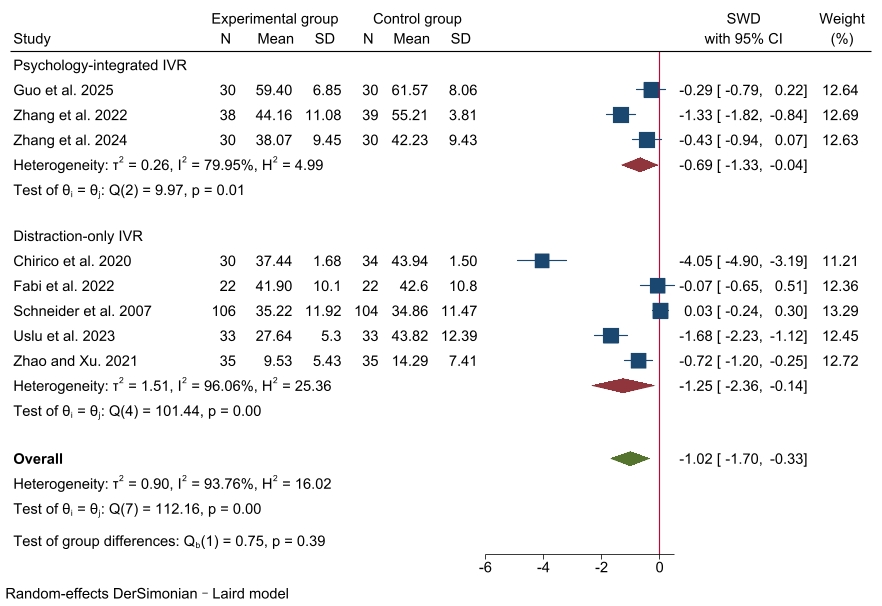


(a)


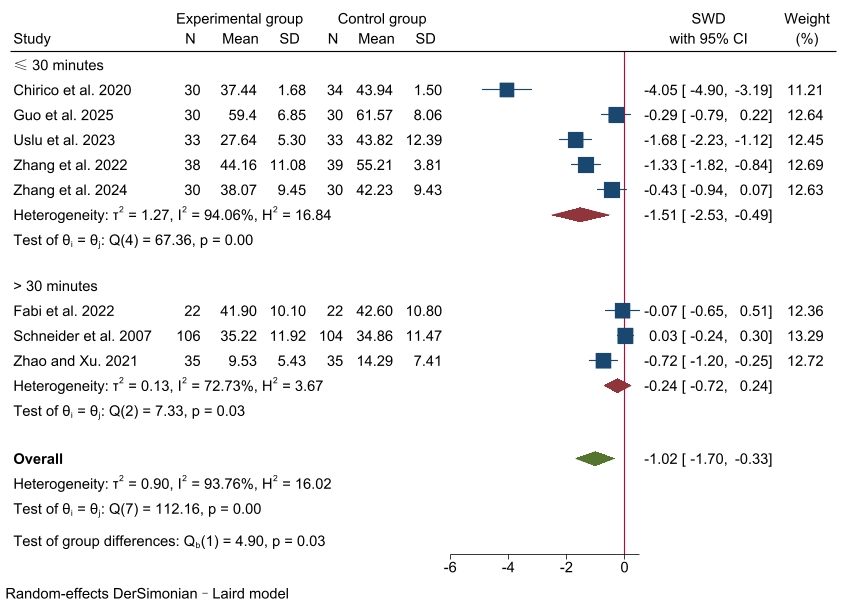


(b)

**Figure S1.** The results of the subgroup analysis for anxiety. (a) Based on intervention content; (b) Based on intervention length.

**
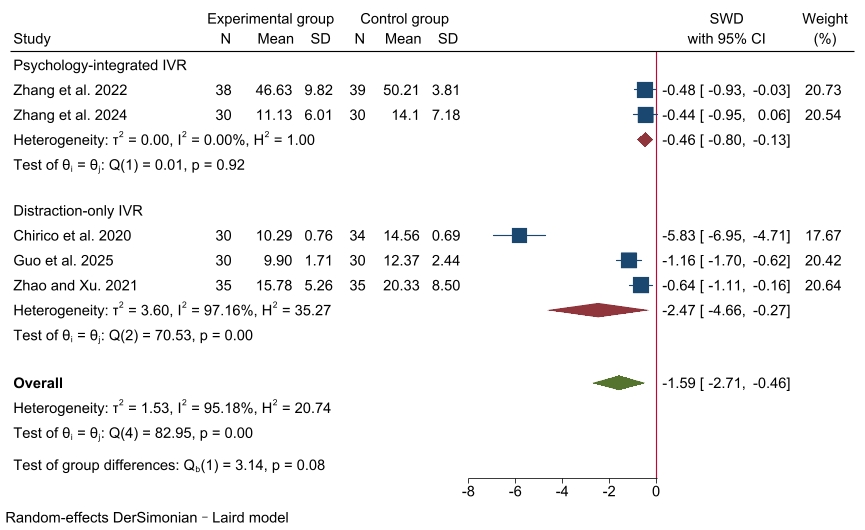
**

(a)


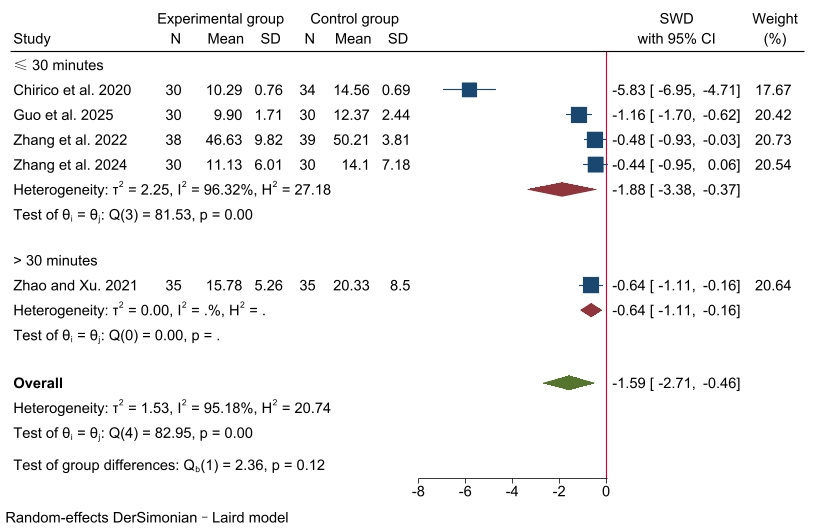
(b)

**Figure S2.** The results of the subgroup analysis for depression. (a) Based on intervention content; (b) Based on intervention length.


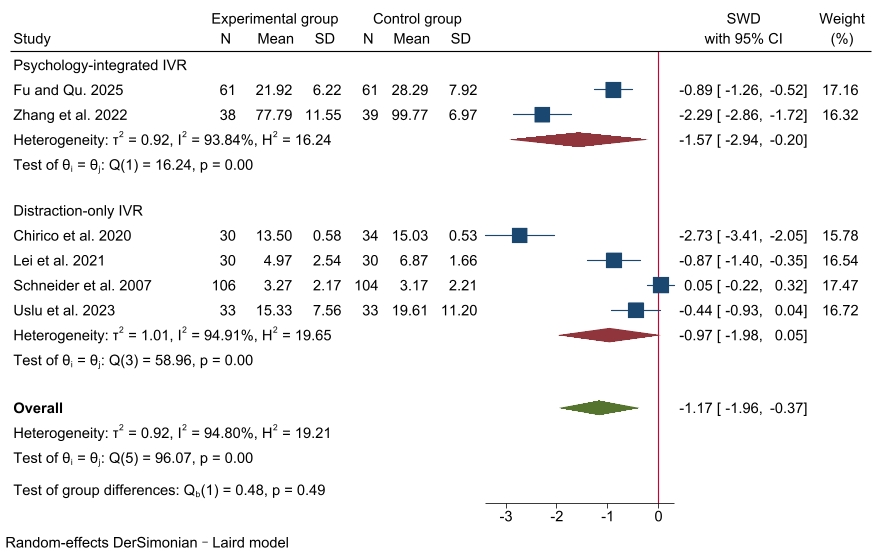
(a)


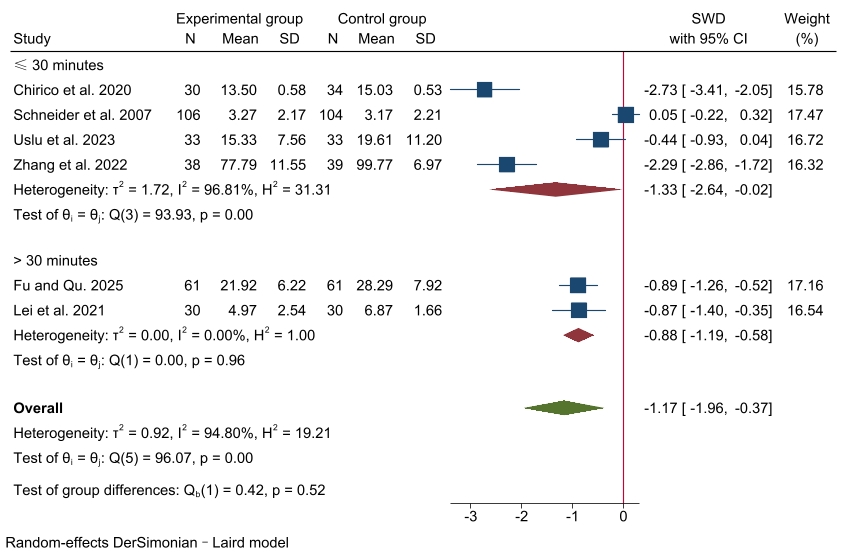
(b)

**Figure S3.** The results of the subgroup analysis for cancer-related fatigue. (a) Based on intervention content; (b) Based on intervention length.


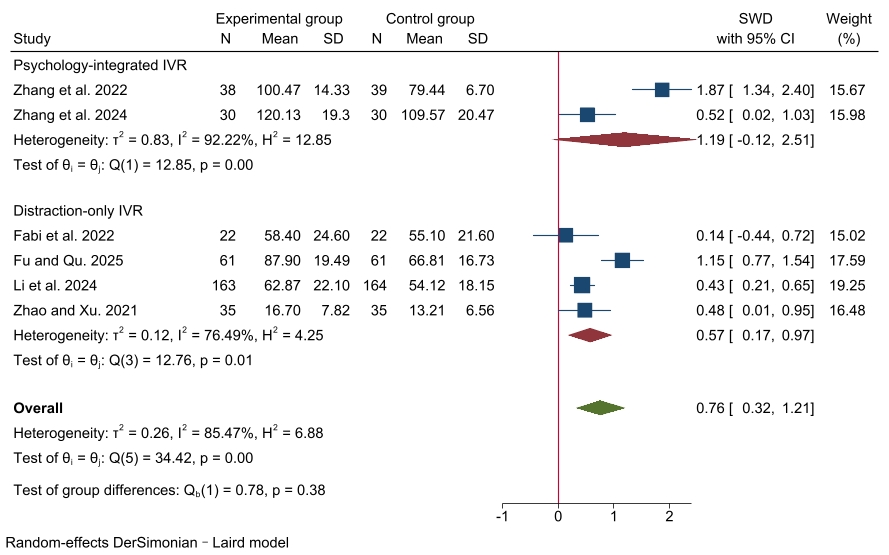
(a)


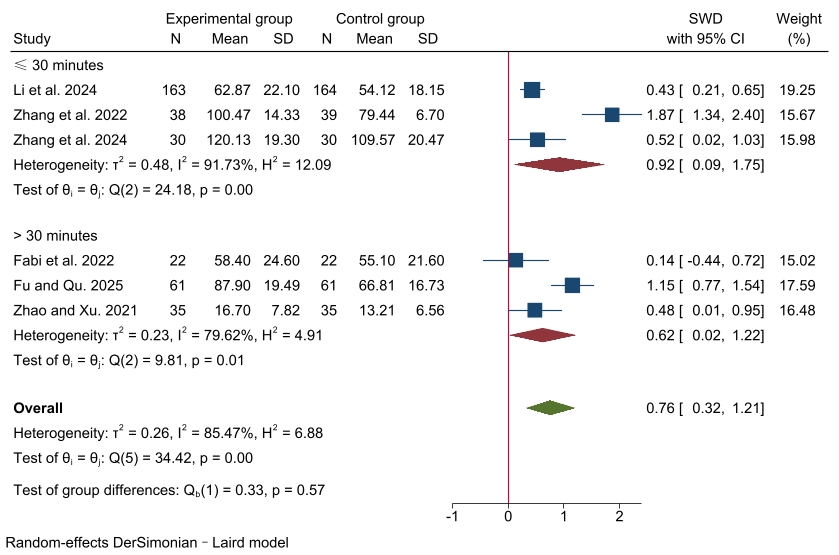
(b)

**Figure S4.** The results of the subgroup analysis for quality of life. (a) Based on intervention content; (b) Based on intervention length.


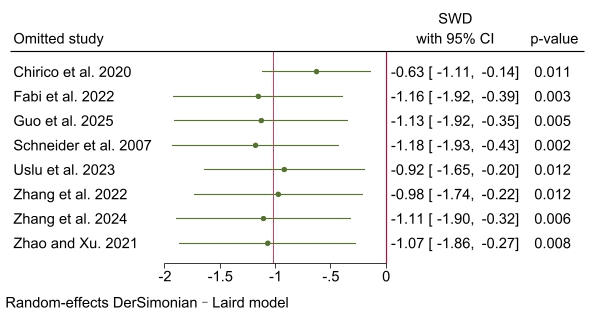


(a)


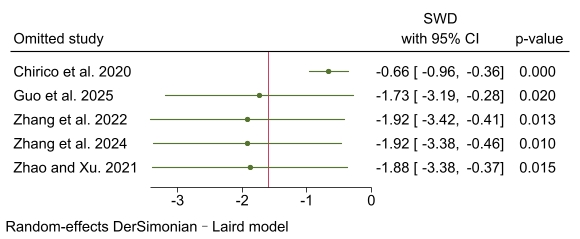


(b)


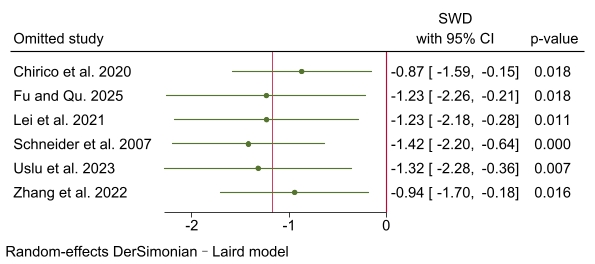
(c)


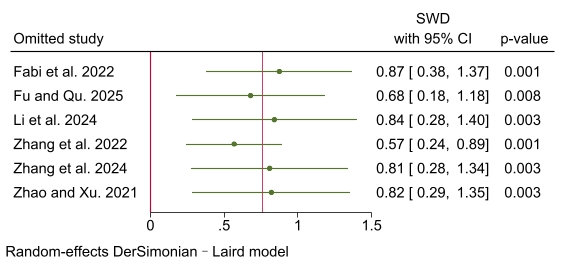


(d)

**Figure S5.** The sensitivity analyses for anxiety, depression, cancer-related fatigue, and quality of life. (a) anxiety; (b) depression; (c) cancer-related fatigue; (d) quality of life.

**Table S2.** GRADE summary of the quality of the evidence for the outcomes.

| Outcome | Quality assessment | | | | | No. of Participants  (studies) | Effect size (95% CI) | Quality of the evidence (GRADE) |
| --- | --- | --- | --- | --- | --- | --- | --- | --- |
|  | Risk of bias | Inconsistency | Indirectness | Imprecision | Other considerations |  |  |  |
| 1. Anxiety | Serious^a^ | Serious^b^ | No serious^c^ | No serious^d^ | None | 751 (8) | SMD = -1.02, [-1.70, -0.33] | ⨁⨁⭘⭘  Low |
| 1. Depression | Serious^a^ | Serious^b^ | No serious^c^ | No serious^d^ | None | 331 (5) | SMD = -1.59, [-2.71, -0.46] | ⨁⨁⭘⭘  Low |
| 1. Cancer-related fatigue | Serious^a^ | Serious^b^ | No serious^c^ | No serious^d^ | None | 599 (6) | SMD = -1.17, [-1.96, -0.37] | ⨁⨁⭘⭘  Low |
| 1. Quality of life | Serious^a^ | Serious^b^ | No serious^c^ | No serious^d^ | None | 700 (6) | SMD = 0.76, [0.32, 1.21] | ⨁⨁⭘⭘  Low |

^a^ Most information is from studies rated as moderate or high risk of bias.

^b^ Heterogeneity in the I^2^ test >50%.

^c^ The PMRE is compared directly with the similar controls.

^d^ The 95% CI excludes pooled effect sizes that are not clinically significant.
